# Detecting *Schistosoma* infections in endemic countries: a diagnostic accuracy study in rural Madagascar

**DOI:** 10.1186/s40249-025-01292-x

**Published:** 2025-03-17

**Authors:** Eva Lorenz, Ravo Razafindrakoto, Pia Rausche, Zaraniaina Tahiry Rasolojaona, Nantenaina Matthieu Razafindralava, Alexandre Zerbo, Yannick Höppner, Heidrun von Thien, Njary Rakotozandrindrainy, Cheick Oumar Doumbia, Philipp Klein, Jean-Marc Kutz, Paul L. A. M. Corstjens, Claudia de Dood, Pytsje T. Hoekstra, Govert J. van Dam, Anna Jaeger, Norbert Georg Schwarz, Egbert Tannich, Mala Rakoto Andrianarivelo, Raphael Rakotozandrindrainy, Rivo Andry Rakotoarivelo, Jürgen May, Tahinamandranto Rasamoelina, Daniela Fusco

**Affiliations:** 1 Department of Infectious Diseases Epidemiology, Bernhard Nocht Institute for Tropical Medicine (BNITM), Bernhard-Nocht-Strasse 74, 20359 Hamburg, Germany; 2 German Center for Infection Research (DZIF), Hamburg-Borstel-Lübeck-Riems, Hamburg, Germany; 3 Institute of Medical Biostatistics, Epidemiology and Informatics, University Medical Centre of the Johannes Gutenberg University Mainz, Mainz, Germany; 4 Centre d’Infectiologie Charles Mérieux, University of Antananarivo, 101 Antananarivo, Madagascar; 5 Research Group: Implementation Research, Bernhard Nocht Institute for Tropical Medicine (BNITM), Bernhard-Nocht-Strasse 74, 20359 Hamburg, Germany; 6 Department of Cellular Parasitology, Bernhard Nocht Institute for Tropical Medicine (BNITM), Bernhard-Nocht-Strasse 74, 20359 Hamburg, Germany; 7 Department of Microbiology and Parasitology, University of Antananarivo, 101 Antananarivo, Madagascar; 8 University Clinical Research Center, University of Sciences, Techniques and Technologies of Bamako, Bamako, Mali; 9 Department of Cell and Chemical Biology, Leiden University Medical Center, Albinusdreef 2, 2333 ZA Leiden, the Netherlands; 10 Department of Parasitology, Leiden University Medical Center, Albinusdreef 2, 2333 ZA Leiden, the Netherlands; 11 National Reference Centre for Tropical Pathogens, Bernhard Nocht Institute for Tropical Medicine (BNITM), Hamburg, Germany; 12 Department of Infectious Diseases, University of Fianarantsoa Andrainjato, 301 Fianarantsoa, Madagascar; 13 Department of Tropical Medicine I, University Medical Center Hamburg-Eppendorf (UKE), Hamburg, Germany

**Keywords:** Schistosomiasis, Diagnostics, Imperfect reference standards, Bayesian latent class models, Prevalence, Madagascar

## Abstract

**Background:**

*Schistosoma haematobium* and *S. mansoni* are endemic in Madagascar, but reliable diagnostic tools are often lacking, contributing to exacerbate transmission and morbidity. This study evaluated the diagnostic accuracy of three tests for schistosome infection in Malagasy adults from areas of medium to high endemicity.

**Methods:**

This cross-sectional study enrolled adults from three primary health care centres in Madagascar. Urine and blood samples were tested for schistosome infection using polymerase chain reaction (PCR), up-converting reporter particle lateral flow for the circulating anodic antigen (UCP-LF CAA), and point-of-care circulating cathodic antigen (POC-CCA) tests. Bayesian latent class models were used to assess diagnostic accuracies and disease prevalence.

**Results:**

Of 1339 participants, 461 were from *S. haematobium* and 878 from *S. mansoni* endemic areas. Test detection rates were 52% (POC-CCA), 60% (UCP-LF CAA), and 66% (PCR) in the *S. haematobium* area, and 54%, 55%, and 59% respectively in the *S. mansoni* area. For *S. haematobium*, PCR and UCP-LF CAA showed high sensitivity (Se, median 95.2% and 87.8%) but moderate specificity (Sp, 60.3% and 66.2%), while POC-CCA performed moderately (Se: 64.5%; Sp: 59.6%). For *S. mansoni*, PCR and POC-CCA demonstrated high diagnostic accuracy (Se > 90%, Sp > 80%), while UCP-LF CAA showed good sensitivity (79.9%) but moderate specificity (69.7%).

**Conclusions:**

While population-level prevalence estimates were similar across tests, individual-level agreement was only low to moderate. Our findings suggest that optimal diagnostic strategies should be tailored to specific endemic settings, continued development of accurate diagnostics suitable for highly endemic settings remains a priority.

**Graphical Abstract:**

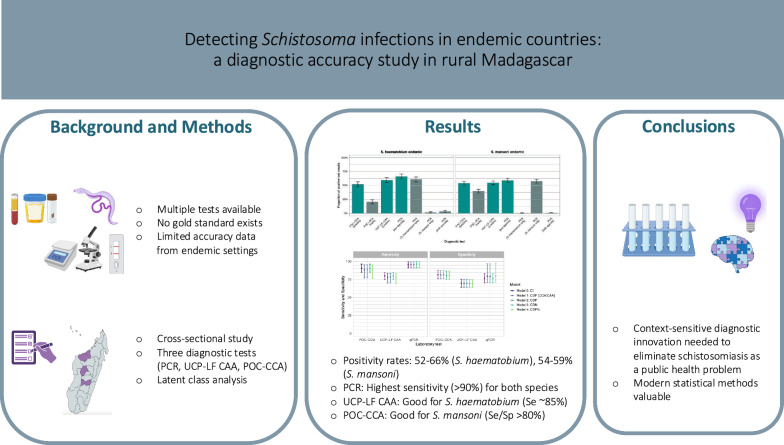

**Supplementary Information:**

The online version contains supplementary material available at 10.1186/s40249-025-01292-x.

## Background

Accurate diagnosis is crucial for the control and elimination of schistosomiasis, yet available diagnostic tools often fail to meet the needs in endemic settings [1–3]. In line with the neglected tropical diseases (NTD) Roadmap 2021–2030, schistosomiasis is targeted for elimination as a public health problem, requiring more sensitive diagnostics for both population screening and individual case management [4]. Schistosomiasis ranks third among NTDs in terms of disability-adjusted life years (DALYs), with up to 2.5 million reported cases [5, 6], and is a major public health and socio-economic challenge in many sub-Saharan African (SSA) countries. Over 700 million people live in endemic areas and are at risk of infection [7, 8] with an estimated 251.4 million requiring preventive treatment in 2021, with approximately 90% of those living in SSA [9, 10]. The disease is caused by *Schistosoma* spp., transmitted to humans through prolonged contact with contaminated fresh water [11]. *S. mansoni* causes the intestinal form of the disease, and *S. haematobium* causes urogenital schistosomiasis [7, 12, 13]. Both can become chronic, leading to serious health conditions such as liver fibrosis, genital schistosomiasis and cancer.

Treatment with praziquantel (PZQ) is safe and efficacious against all schistosome [12, 14, 15]. Lower cure rates have been reported in numerous areas [16, 17], and understanding PZQ resistance is hampered by the lack of sensitive and accurate *Schistosoma* detection tools. While mass drug administration (MDA) is widely used to control schistosomiasis in endemic areas [14, 18], the precise extent of disease burden remains unclear [7, 19]. The global burden of schistosomiasis is estimated at 1.9 million DALYs [6, 20–22]. MDA programmes alone are not enough to eliminate schistosomiasis. WHO has therefore provided guidance and advocacy on non-medical interventions to improve control [23, 24].

Current diagnosis of schistosomiasis is based on eggs in stools or urine, depending on the species involved. WHO recommends microscopy of three urine or stool samples collected over three consecutive days as reference standard [23]. While microscopy is cost-effective [25–28], its sensitivity can be low, especially for low-intensity infections. In addition, the collection of multiple samples is logistically challenging. This study evaluates the three diagnostic approaches, i.e., point-of-care circulating cathodic antigen (POC-CCA), up-converting reporter particle lateral flow circulating anodic antigen (UCP-LF CAA), and real-time polymerase chain reaction (PCR). POC-CCA has been validated for *S. mansoni* diagnosis, with sensitivity (Se) of 60% to 90% depending on infection intensity and endemicity [29]. WHO recommends it for population-based prevalence assessment in *S. mansoni* endemic areas [23], though its performance for *S. haematobium* remains variable [30]. UCP-LF CAA [31], has demonstrated promising results across multiple settings [32–34], with specificities (Sp) exceeding 95% [35]. PCR-based diagnosis, with serum-based detection offers high Se for both species [36]. Practical implementation in endemic settings faces infrastructure and resource challenges. Systematic evaluation of these methods in endemic settings is essential to reduce morbidity and progress towards elimination and enabling fit-for purpose application of available tools [37, 38].

Schistosomiasis in Madagascar affects over 50% of the population. *S. haematobium* occurs in the western and northern half of the island, while *S. mansoni* in the eastern and southern parts and the central highlands [39, 40]. Co-infections occur in the north-central and south-western regions [41]. The national program is mostly based on MDA. Even though MDAs are repeated frequently with a good territorial coverage, the country is lagging behind schedule for the adaptation of the national guidelines to those recently released by the WHO [42]. The heavy centralization of the health system so as the difficulties of accessing health services by the Malagasy population, creates strong barriers for the adaptation of schistosomiasis related services. Over 60% of the population lives more than five kilometres from the nearest health care centre [43] and in 2021, it was reported that Madagascar had a healthcare worker density of less than 0.5 per 1000 inhabitants [44].

This study investigates the diagnostic accuracy of three candidate diagnostic tests (Table 1, characteristics of UCP-LF CAA and POC-CCA adapted from [37]) for use in endemic settings. We selected the POC-CCA, UCP-LF CAA and PCR for the detection of *Schistosoma* infection in individuals attending primary health care facilities in Madagascar. These tests could fill diagnostic gaps for *Schistosoma* detection at different levels of the healthcare system. We used Bayesian latent class models (BLCM) to assess diagnostic accuracy [45] estimating prevalence, Se, and Sp for each tests.

**Table 1:** Description of the three laboratory tests assessed

Diagnostic	Sample type	Ease of use	Cost of test	Where assessed	Metric	Definition of positive
POC-CCA	Urine	Field-applicable, rapid	Low	PHCCs in Madagascar	Intensity of band against metric	G4–G10
UCP-LF CAA	Urine	Minimal laboratory facilities	Moderate to high	Laboratory in Madagascar	Antigen concentrations in pg/ml	Cut-off of ≥ 2 pg/ml
PCR	Serum	Sophisticated laboratory facilities	High	Laboratory at BNITM Hamburg	Cycle threshold values	Standard curve

*BNITM* Bernhard Nocht Institute for Tropical Medicine, *POC-CCA* point-of-care circulating cathodic antigen, *PCR* polymerase chain reaction, *PHCC* primary health care centre, *UCP-LF CAA* up-converting reporter particle lateral flow circulating anodic antigen

## Methods

### Primary dataset details: study design, setting and participants

The cross-sectional diagnostic accuracy study enrolled adult patients at primary health care centres (PHCC) in Madagascar between March 2020 and January 2021 based on consecutive sampling. Specifically, the sample collection took place in Andina (20°30′58.8ʺS, 47°09′05.2ʺE) and Tsiroanomandidy (18°46′21ʺS, 46°02′57ʺE) in the centre, Ankazomborona (16°06′50ʺS, 46°45′24ʺE) in the north western part of the island. The centres were selected based on their location in areas suspected to be endemic for *S. mansoni* (Andina and Tsiroanomandidy) or *S. haematobium* (Ankazomborona) respectively, and their accessibility for study implementation [19, 46]. Participants were eligible for inclusion if they were at least 18 years of age in absence of fever, history of transfusion or congenital anaemia and epileptic or convulsive episodes and after written informed consent was obtained. Participants with a transfusion or congenital anaemia were excluded to limit interference with co-infections and/or genetic factors that might alter the test performances, participants with epileptic or convulsive episodes were excluded to limit risk of treatment with PZQ due to possible co-infections with *Tenia solium*. Participants’ basic sociodemographic and clinical characteristics (date of admission, sex, age, area of residence and presenting symptoms) were recorded in REDCap. We excluded individuals treated with PZQ within 12 months prior to enrolment to rule out that PCR could detect cured non-active infections. All participants were tested using the three tests under evaluation. Consequently, the diagnostic data represented a sample drawn from this target population that informed the estimation of prevalence and Se and Sp of the tests. Participants who were scored as trace or positive by POC-CCA received immediate treatment with PZQ.

### Sample collection and processing

POC-CCA analysis was performed by a trained laboratory technician on a single urine sample taken on the day of recruitment. Tests were scored using the G-scores and grouped into three categories: negative, trace, and positive. For the UCP-LF CAA analysis, a sample of urine (a maximum of 25 ml) was taken from each participant and stored locally at room temperature for a maximum of seven days until transport to the central laboratory to reduce sample deterioration. On receipt at the central laboratory (Charles Mérieux infectiology centre, Antananarivo), samples were aliquoted for long-term storage at – 80 °C. For the PCR analyses, a sample of 9 ml of venous blood was collected in serum separating tubes from each participant. The blood samples were centrifuged at 1600 × *g* for 10 min and two aliquots of 1 ml serum each were prepared by qualified laboratory technicians. The samples were stored at – 20 °C according to the required quality standards and transferred to long-term storage at − 80 °C in Madagascar. One of the serum aliquots was shipped to Hamburg on dry ice and stored at − 80 °C until UCP-LF CAA testing.

### Diagnostic test methods

#### Target condition

The schistosome infection status was determined by three laboratory tests (i.e., POC-CCA, UCP-LF-CAA, PCR). In other words, we considered the observed data of the three tests as indicators of an underlying, not directly observable variable (i.e., *S. haematobium* or *S. mansoni* active infection). Each participant had one result per test.

#### Point-of-care circulating cathodic antigen (POC-CCA)

A commercial immunochromatographic lateral flow POC test (Rapid Medical Diagnostics, South Africa—batch number: 200326039) was used to test urine for the presence of CCA of *Schistosoma* spp. and was performed using the G-scores [47]. The highest CCA concentrations are detected in *S. mansoni* infections, which makes the test particularly suitable for the diagnosis of intestinal schistosomiasis. The concentrations in urogenital schistosomiasis (*S. haematobium*) are variable, and also appear to differ from region to region. In general, there is a link between the Se of the test and the intensity of the *S. haematobium* infection. Some moderate to severe infection with *S. haematobium* can be diagnosed with the CCA urine test strip though the test lacks Se for mild *S. haematobium* infections. Briefly, the assay uses only two drops of urine into a cassette. The test was read after 20 min and the band density was recorded according to a semi-quantitative scoring system on a scale of 1–10 (G-scores) and divided into three categories: negative, trace and positive [47–49]. Samples were considered positive if the CCA score was equal or higher than the cut-off of G4. An internal quality control was included to validate the cut-off of the POC-CCA (G4). No clinical information was provided to the performers or readers of the test.

#### Up-converting reporter particle lateral flow circulating anodic antigen (UCP-LF CAA) assay

The UCP-LF-CAA, a non-commercially available assay, detects a *Schistosoma* genus-specific antigen originating from the gut of the adult worm. The urine samples were pre-treated with tri-chloroacetic acid and after centrifugation, the clear supernatants were concentrated by ultracentrifugation. The samples were then incubated with the monoclonal UCP antibody conjugate, then LF strips were added to the solution and left to run and dry overnight. UCP-LF CAA analysis was performed on a urine sample on the day. For quantitative measurement of bound CAA, the strips were scanned by an UCP strip reader and results analysed with a special software [31]. The materials used in the UCAAhT417 format of the UCP-LF CAA diagnostic test have a lower QC limit of 2 pg/ml when performing a single test. Samples were considered positive if the CAA concentration exceeded the pre-specified cut-off of 2 pg/ml implying high clinical Sp [31]. No clinical information was provided to the performers or readers of the test.

#### Polymerase chain reaction (PCR)

Semi-quantitative standardised PCR was performed on serum to detect a species-specific cell free circulating deoxyribonucleic acid (DNA) derived from viable schistosomula, adult parasites or disintegrated eggs [50]. The analyses were performed in the laboratory of the Bernhard Nocht Institute for Tropical Medicine, in Hamburg, Germany. The PCR analysis was based on the previously published protocol of Frickmann et al. [36], targeting *Dra1* sequence of the *S. haematobium* complex and the *Sm1-7* sequence of the *S. mansoni* complex. Specifically, the primers used for the amplification were: *S. mansoni*—Forward Primer: 5′ CAA CCG TTC TAT GAA AAT CGT TGT 3′, *S*. *mansoni*—Reverse Primer: 5′ CCA CGC TCT CGC AAA TAA TCT 3′, *S*. *mansoni*—Probe: ‘Fam-TCC GAA ACC ACT GGA CGG ATT TTT ATG AT-BHQ1’, *S*. *haematobium*—Forward Primer: 5′ GAT CTC ACC TAT CAG ACG AAA C 3′, *S. haematobium*—Reverse Primer: 5′TCA CAA CGA TAC GAC CAA C 3′, *S. haematobium*—Probe: 5′ Joe-TGT TGG AAG ZGC CTG TTT CGC AA-BHQ1 3′ all synthesized by Biomers.net, Ulm, Germany. Primers and a probe were added for the detection of Phocid herpesvirus (PhHV) DNA as internal positive control (PhHV—Forward Primer: 5′ GGG CGA ATC ACA GAT TGA ATC 3′, PhHV—Reverse Primer: 5′ GCG GTT CCA AAC GTA CCA A 3′, PhHV—Probe: 5′ Cy5.5-TTT TTA TGT GTC CGC CAC CA-BBQ 3′). All results with a clean sigmoid curve within the PCR cycles were considered positive against a pre-specified threshold [36, 51]. No clinical information was provided to the performers or readers of the test.

### Latent class model specification

Assessing the accuracy of tests for schistosomiasis infections is challenging as there is no gold standard. Traditional approaches that assume a perfect reference standard can lead to biased accuracy estimates. While Composite Reference Standards (CRS) are valuable tools when appropriately implemented, they too have limitations when treated as perfect references. To address these challenges, we employed Latent Class Models (LCM), a statistical framework that explicitly accounts for the imperfect nature of all tests under evaluation [52]. Our Bayesian implementation of these models (BLCM) offers additional advantages by allowing the incorporation of prior knowledge from previous studies and appropriately handling the complex uncertainty inherent in evaluating multiple imperfect tests simultaneously. While this approach can provide less biased estimates of prevalence and test accuracy than conventional methods, it must be acknowledged that these methods are considered methodologically complex and difficult to validate [45].

#### Diagrammatic representation

**Fig. 1:**
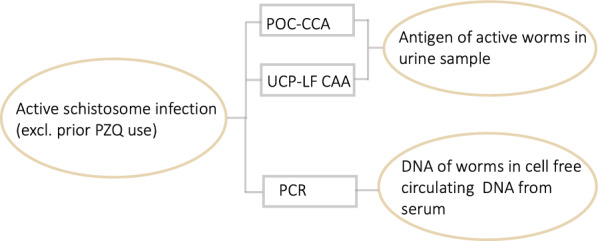
Heuristic model. The model shows the assumed relationships between latent class (oval) and diagnostic test results (rectangles). All measures are worm-based. Abbreviations: *DNA* deoxyribonucleic acid, *POC-CCA* point-of care circulating cathodic antigen, *PCR* polymerase chain reaction, *PZQ* praziquantel, *UCP-LF CAA* up-converting reporter particle lateral flow circulating anodic antigen

We first created a heuristic diagram for schistosome infection to illustrate our assumptions about the relationships between the observed test results and schistosome infection (Fig. 1). This diagram identifies the measurand of each test, i.e., the quantity it measures. POC-CAA, UCP-LF CAA, and PCR use different techniques to measure (i) antigen of active worms in urine samples (POC-CCA and UCP-LF-CAA) and (ii) the presence of parasitic worms in cell free circulating DNA from serum (PCR). While all three tests detect products of active worm infection, they differ in their biological mechanisms: PCR detects DNA released from worms into circulation, while POC-CCA and UCP-LF CAA detect specific antigens excreted by worms that appear in urine. POC-CCA and UCP-LF CAA are both antigen tests that target different antigens and were performed on the same urine sample. The underlying semi-quantitative POC-CCA values and the UCP-LF CAA antigen concentrations may be positively correlated and are both influenced by the intensity of the infection (worm load/burden). While the relationship between worm burden and the amount of detectable circulating DNA is not fully understood, all three tests might produce false negative results particularly in individuals with low infection intensity, potentially leading to correlated errors in test outcomes. Based on our methodological considerations and biological understanding of the tests’ mechanisms, four probable correlations between diagnostic tests were explored hypothesising that underlying worm burden may lead to a conditional dependence between either two or all three test results, even if the tests are based on different mechanisms.

#### Statistical model

The observed diagnostic test results were assumed to be results from the two underlying latent classes; (1) schistosome infection measure-positive, and (2) schistosome infection measure-negative. In other words, we considered the observed data of the three diagnostic tests as indicators of an underlying, not directly observable variable (i.e., *S. haematobium* or *S. mansoni* infection). The unknown parameters of the model were the prevalence of the two latent classes and each test’s Se and Sp with respect to its measure.

### Bayesian model estimation

We used a Bayesian approach to fit the LCM (BLCM) to estimate prevalence, Se, and Sp [53]. All parameters were estimated with 95% credible intervals using Stan version 2.32.2 (Stan Development Team, New York, NY, USA) and R version 4.4.1 (R Foundation for Statistical Computing, Vienna, Austria). As the posterior distributions of the parameters of interest (Se, Sp, prevalence) could not be computed analytically, we sampled from the posterior distributions using a Markov Chain Monte Carlo approach with the rstan package through Rstudio (Version 2023.09.0 + 463, R Version 4.3.1). All point estimates reported are posterior medians with corresponding 95% credible intervals, unless otherwise specified. For the main models, non-informative priors were used for all models with truncated prior distributions for the non-specific tests’ Se and Sp to contain them above 40% and avoid label switching (mirror solutions). Model estimates from alternative models using informative priors for UCP-LF CAA and PCR Sp and their specification can be found in section SI2.1 and SI2.3 (see Supplementary Information SI). The Supplement contains details of model specifications, sampling, and model checking. The BLCM models varied from a model assuming conditional independence between all tests (Model 0) to those considering conditional dependence between a single pair of antigen tests using fixed effects (Model 1), those that use random effects to represent dependence between all tests within an infection class (Models 2 and 3) and finally a model that simultaneously accounted for conditional dependence between all three tests among those individuals truly infected and those individuals truly not infected using random effects (Model 4). A class of fixed and random effect models described by Dendukuri and Joseph were used to take account for conditional dependence between tests [54, 55]. Individuals with invalid or missing test results were assumed to be missing at random though excluded from analyses [invalid and missing results were 35 out of 1374 (2.5%)] i.e., complete case analyses were performed. Bayesian leave one out estimate of the expected log pointwise predictive density between two models were used to compare the models.

### Additional considerations

The number and percentage of patients along with descriptive statistics [i.e., median, interquartile range (IQR), minimum and maximum] are reported for continuous non-normally distributed data. Categorical variables (i.e., gender, age groups, and treatment information) have the number and percentage of patients reported. PCR cycle threshold (Ct) values and UCP-LF CAA antigen concentrations are displayed and compared using boxplots with descriptive statistics stratified by three POC-CCA categories (i.e., negative, traces, positive). Proportions of positive test results were calculated as percentages of positive results among all valid tests, with exact binomial 95% confidence intervals (*CI*s). Agreement between tests is assessed using Cohen’s and Fleiss’s kappa statistics, percentage agreements and Prevalence-Adjusted and Bias-Adjusted Kappa (PABAK). To categorise the diagnostic accuracy of the laboratory tests, we defined sensitivities and specificities ≥ 90% as high, as commonly used in clinical research to ensure a high level of reliability and clinical utility [56]. For assessing agreement between tests, we used the following kappa (*κ*) interpretation scale: *κ* < 0: poor agreement; 0 < *κ* < 0.20: slight; 0.20 < *κ* < 0.40: fair; 0.40 < *κ* < 0.60: moderate; 0.60 < *κ* < 0.80: substantial. Spearman's rank correlation coefficients (*ρ*) with 95% *CI*s were calculated to assess the relationships between diagnostic test results. Correlations were calculated excluding extreme CAA values (> 1000 pg/ml) and negative values. Negative UCP-LF CAA concentrations were imputed by a fixed value of 0.5 × cut-off of 2 pg/ml to facilitate visualisation in boxplots on a log-scale [57]. For PCR Ct values, correlations were inversed to reflect that lower Ct values indicate higher parasite loads. Confidence intervals were calculated using Fisher’s Z-transformation. All correlations were computed separately for samples from *S. haematobium* and *S. mansoni* endemic regions, including only samples with positive PCR results and detectable CAA levels (> 2 – < 1000 pg/ml).

### Sample size

The sample size estimation was informed by two key considerations: (1) the precision required for diagnostic accuracy parameters and (2) the requirements for stable Bayesian Latent Class Analysis (BLCA) model convergence. For diagnostic accuracy estimation, we targeted a precision of ± 5% for Se and Sp, assuming expected performances of 85% for *S. haematobium* and 90% for *S. mansoni* tests at an anticipated prevalence of 50%. This indicated minimum required sample sizes of 400 participants for *S. haematobium* and 550 for *S. mansoni* sites. For the BLCA modelling, we followed recommendations suggesting that stable parameter estimation requires larger sample sizes, typically *n* > 450 per site when analysing three diagnostic tests simultaneously [58]. The final achieved sample sizes satisfied both requirements.

## Results

### Participants

**Fig. 2:**
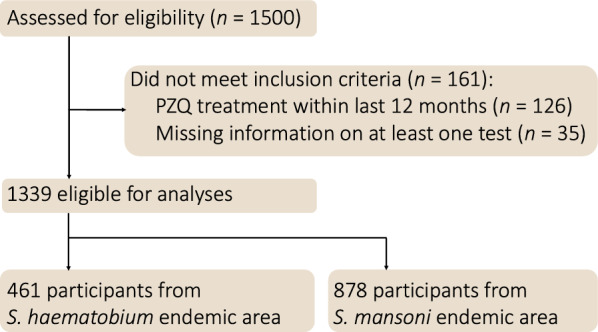
Participant recruitment and selection for diagnostic accuracy evaluation of schistosomiasis tests in Madagascar. From 1500 initially assessed participants, 1339 were eligible for analysis after excluding those with recent praziquantel (PZQ) treatment or missing test results. The final sample comprised 461 participants from *S. haematobium* and 878 from *S. mansoni* endemic areas. *PZQ* praziquantel

Of the 1500 participants recruited, 161 were excluded because of PZQ treatment within 12 months prior to survey and sampling (*n* = 126), or because at least one of the diagnostic tests results was missing (*n* = 35). A total of 1339 participants were eligible for analyses, of whom 461 participants were from a *S. haematobium* (urogenital) and 878 from a *S. mansoni* (intestinal) endemic area (Fig. 2). Samples and diagnostic test results from all three tests under consideration were available for these participants. In the *S. haematobium* endemic area*,* median age was 28 years (IQR: 21 to 40) and 255 (55%) of 461 participants were female. In the *S. mansoni* endemic area*,* median age was 38 years (IQR: 26 to 50) and 473 (54%) of 878 participants were female (Supplementary Table SI1.1; for characteristics of the sample).

### Diagnostic test results

**Fig. 3:**
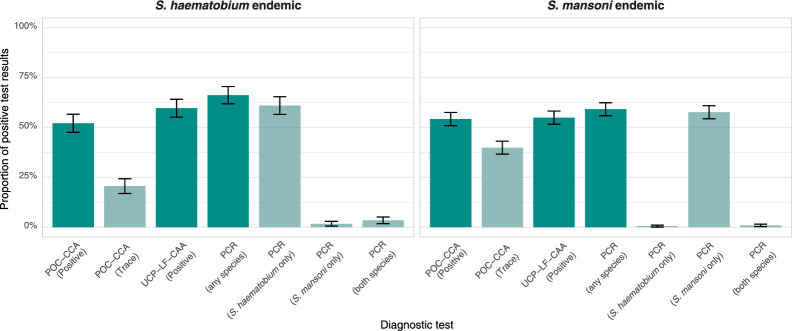
Proportion of positive test results expressed as percentages by each of the three diagnostic tests among the assessed individuals in *S. haematobium* (left) and *S. mansoni* (right) endemic areas. Lighter shades indicate trace results for POC-CCA and species-specific results for PCR. Error bars represent 95% confidence intervals

Detection rates ranged from 52% (POC-CCA) over 60% (UCP-LF CAA) to 66% (PCR) in the *S. haematobium* endemic area. In the *S. mansoni* endemic area, detection rates ranged from 54% (POC-CCA) over 55% (UCP-LF-CAA) to 59% (PCR) (Fig. 3, for details see Supplementary Information I Table SI1.2.).

### Test result patterns

**Fig. 4:**
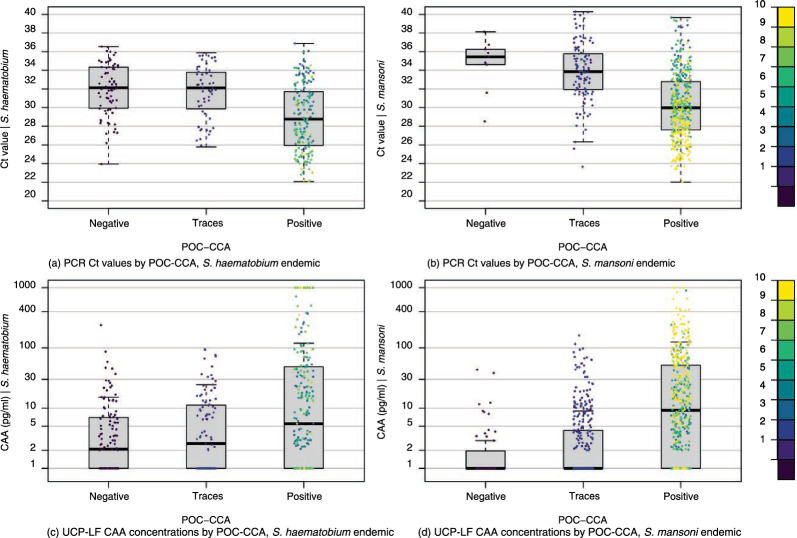
Distribution of PCR Ct values and UCP-LF CAA concentrations measured in pg/ml displayed by three POC-CCA categories (i.e., negative, traces, positive). Subfigures show (**a**) PCR Ct values by POC-CCA, *S. haematobium* endemic, **b** PCR Ct values by POC-CCA, *S. mansoni* endemic, **c** UCP-LF CAA concentrations by POC-CCA, *S. haematobium* endemic, **d** UCP-LF CAA concentrations by POC-CCA, *S. mansoni* endemic. The colour gradient indicates POC-CCA G score grading. *Ct* cycle threshold, *PCR* polymerase chain reaction, *pg/ml* picograms per millilitre, *POC-CCA* point-of-care circulating cathodic antigen, *UCP-LF CAA* up-converting reporter particle lateral flow circulating anodic antigen

Figure 4 shows the distribution of PCR Ct values and UCP-LF CAA concentrations across POC-CCA categories in *S. haematobium* and *S. mansoni* endemic areas. In both settings, trace results showed distributions more similar to negative than positive cases. Based on this observation, trace results were classified as negative for subsequent dichotomous analyses.

**Fig. 5:**
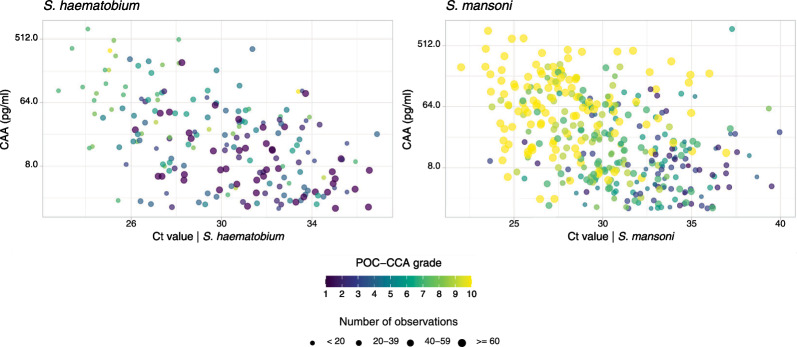
Relationship between diagnostic test results for *S. haematobium* and *S. mansoni* infections. Scatter plots show the relationship between PCR cycle threshold (Ct) values, UCP-LF-CAA concentrations (pg/ml), and POC-CCA grades. UCP-LF CAA antigen concentrations that were either imputed by a fixed value of 0.5 × cut-off of 2 pg/ml or the maximum values of 1000 were excluded from this visualisation to ease interpretation. Point sizes indicate the frequency of observations (< 20, 20–39, 40–59, ≥ 60 observations). POC-CCA grades range from 1 (negative) to 10 (strongly positive), represented by the color gradient. Data are shown separately for samples from *S. haematobium* endemic regions (left) and *S. mansoni* endemic regions (right). Note the logarithmic scale on the y-axis

The relationship between diagnostic test results showed distinct patterns across endemic settings. In *S. mansoni* endemic sites, a clear trend was observed with higher POC-CCA grades (yellower colors) clustering in the upper left quadrant, indicating stronger correlation between lower PCR Ct values (higher parasite loads) and higher CAA concentrations (Fig. 5). This pattern was particularly evident for samples with high frequency (larger dots). In contrast, the *S. haematobium* endemic site showed a less pronounced relationship, with more scattered distribution of POC-CCA grades across the Ct value range. In both settings, CAA concentrations generally increased (shown on the log-scale y-axis) as PCR Ct values decreased, though this inverse correlation appeared stronger in the *S. mansoni* endemic sites. The size of data points reveals that the majority of observations fell within intermediate frequency ranges (20–59 observations per point).

Moderate positive correlations were observed for pairwise comparisons of the three diagnostic methods in the *S. haematobium* endemic setting, while moderate to strong positive correlations were observed in the *S. mansoni* endemic setting (details in Supplementary Information I). In the *S. haematobium* endemic region, the strongest correlation was found between UCP-LF CAA and PCR (*ρ* = 0.48, 95% *CI:* 0.36–0.58), followed by POC-CCA and PCR (*ρ* = 0.41, 95% *CI:* 0.28–0.52), and POC-CCA and UCP-LF CAA (*ρ* = 0.40, 95% *CI:* 0.28–0.52). In the *S. mansoni* endemic region, the strongest correlation was found between POC-CCA and PCR (*ρ* = 0.60, 95% *CI:* 0.53–0.66), followed by POC-CCA and UCP-LF AG (*ρ* = 0.52, 95% *CI:* 0.44–0.59), and UCP-LF CAA and PCR (*ρ* = 0.44, 95% *CI:* 0.35–0.52).

Table 2 presents the frequency of diagnostic test result combinations across three different methods (POC-CCA, UCP-LF-CAA, and PCR) in *S. haematobium* and *S. mansoni* endemic areas. Complete agreement of all three tests was observed in 41% of cases in the *S. haematobium* area (29% all positive, 12% all negative) and 57% in the *S. mansoni* area (35% all positive, 22% all negative). The remaining cases showed various patterns of discordant results between the three diagnostic methods. Further details on the pairwise and overall agreement are provided in Supplementary Table SI1.2 and SI1.3.

**Table 2:** Observed frequency of each response profile with overall percentage agreement shaded in gray

Response profile			Observed frequency	%	Observed frequency	%
POC-CCA	UCP-LF-CAA	PCR				
			*S. haematobium* endemic area		*S. mansoni* endemic area	
0	0	0	54	12	195	22
1	0	0	44	10	47	5
0	1	0	37	8	85	10
1	1	0	21	5	32	4
0	0	1	48	10	66	8
1	0	1	40	9	88	10
0	1	1	82	18	56	6
1	1	1	135	29	309	35

Zeros (0) indicate negative test results and Ones (1) indicate a positive test result. Observed frequency shown corresponds to three test results from 461 participants from *S. haematobium* endemic area and 878 participants from *S. mansoni* endemic area

### Bayesian latent class analysis: estimated diagnostic accuracies and prevalence

**Fig. 6:**
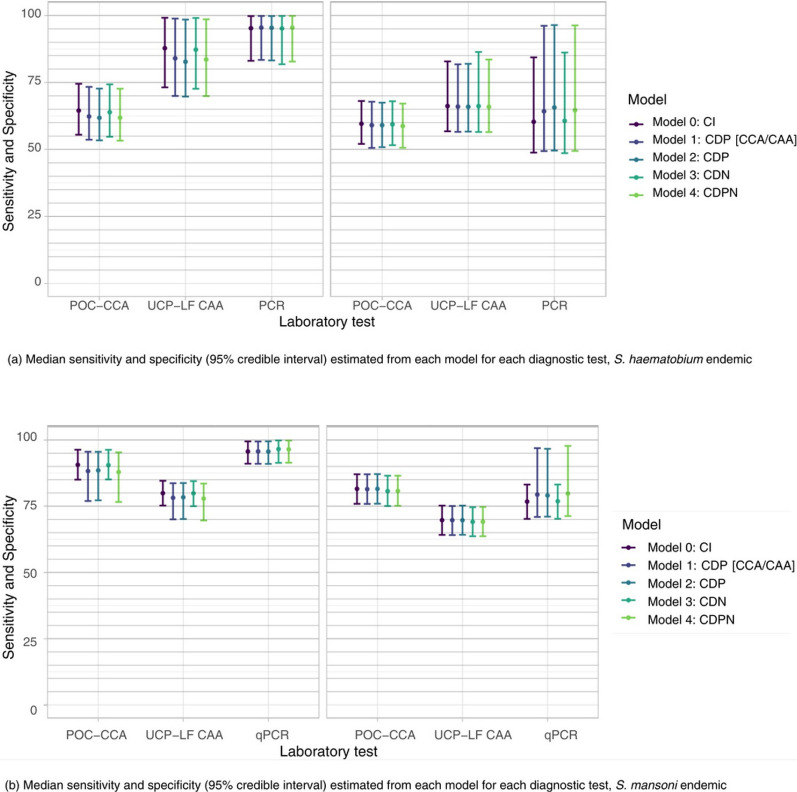
Diagnostic accuracy of the three tests under investigation based on Bayesian Latent Class Analysis in (**a**) *S. haematobium* and (**b**) *S. mansoni* endemic regions. *CI* conditional independence, *CDP* conditional dependence in positives, *CDN* conditional dependence in negatives, *CDPN* conditional dependence in positives and negatives, *PCR* polymerase chain reaction, *POC-CCA* point-of-care circulating cathodic antigen, *UCP-LF-CAA* up-converting reporter particle lateral flow circulating anodic antigen

Figure 6 presents Se and Sp estimates from five BLCM for the three diagnostic tests in *S. haematobium* and *S. mansoni* endemic areas [median values and 95% credible intervals (CrI)]. In both settings, PCR showed the highest sensitivity (95.2%, 95% CrI: 83.1–99.8 for *S. haematobium*; 95.7%, 95% CrI: 91.0–99.5 for *S. mansoni*) with narrow CrI, followed by UCP-LF CAA (87.8%, 95% CrI: 73.2–99.1) and POC-CCA in *S. mansoni* areas (90.6%, 95% CrI: 85.0–96.3). POC-CCA demonstrated lower sensitivity in *S. haematobium* (64.5%, 95% CrI: 55.5–74.5) compared to *S. mansoni*. Specificity was consistently high for POC-CCA (81.5%, 95% CrI: 75.9–87.1), while both PCR and UCP-LF CAA showed moderate specificity (76.7%, 95% CrI: 70.2–83.2 and 69.7%, 95% CrI: 64.2–75.2 respectively). Notably, the different assumptions about conditional dependence between tests (Models 0–4) yielded similar estimates with overlapping credible intervals, suggesting robust results regardless of the dependency structure assumed. Prevalence of *S. mansoni*, and *S. haematobium* were estimated as 49.5% (95% CrI: 43.7–55.4), and 48.1% (95% CrI: 34.4–67.8), respectively. Note that these are not population-level estimates, but rather prevalence estimates among participants recruited at the PHCCs. Details on Se, Sp, negative and positive predictive values and prevalence can be found in Supplementary Tables SI2.1–SI2.2 along with model comparisons (Table SI2.3). Sensitivity analyses using informative priors for test specificities and alternative trace result interpretation (Supplement 2.3, 2.4) confirmed the robustness of our findings regarding test performance characteristics. While prevalence estimates were sensitive to trace result interpretation, particularly in *S. mansoni* endemic areas (increasing from ~ 50% to ~ 77% when interpreting traces as positive), the relative performance patterns of the diagnostic tests remained stable across all model specifications.

#### Test performance in the *S. haematobium* endemic region

For POC-CCA, Se and Sp were low (64.5%, 95% CrI: 55.5–74.5) and Sp (59.6%, 95% CrI: 52.1–68.1 respectively), in line with the caveat that the use of POC-CCA for detecting S. *haematobium* infections performs poorly. Imperfect Se prevented infection rule-out in combination with an imperfect Sp preventing infection rule-in, leaving an unacceptably high uncertainty in the validity of results. UCP-LF CAA and PCR Se were substantial (87.8%, 95% CrI: 73.2–99.1) and high (95.2%, 95% CrI: 83.1–99.8), respectively, but imperfect Sp prevented disease rule-in, and consequently misclassified a considerable proportion of non-infected participants as infected.

#### Test performance in the *S. mansoni* endemic region

POC-CCA Se and Sp were high (90.6%, 95% CrI: 85.0–96.3) and substantial (81.5%, 95% CrI: 75.9–87.1), respectively, enabling acceptable infection classification. UCP-LF CAA Se was substantial (79.9%, 95% CrI: 75.2–84.6), but imperfect Sp prevented infection rule-in, and consequently misclassified a considerable proportion of non-infected participants as infected. PCR Se and Sp were high (95.7%, 95% CrI: 91.0–99.5) and substantial (76.7%, 95% CrI: 70.2–83.2), enabling acceptable infection classification.

## Discussion

Producing correct estimates of disease prevalence and diagnostic test accuracy is challenging without a perfect reference standard. We used BLCM to estimate multiple tests’ accuracies for the detection of two species of *Schistosoma*, along with disease prevalence.

### Summary of findings

The main implication from our study is that no single laboratory test is universally optimal for detecting *Schistosoma* infection. Our findings revealed distinct patterns for *S. haematobium* and *S. mansoni* endemic areas. In an *S. haematobium* area, for which effective diagnostics other than microscopy are scarce, PCR and UCP-LF CAA showed good Se but limited Sp, while POC-CCA performed poorly overall. In contrast, for *S. mansoni*, both PCR and POC-CCA demonstrated high diagnostic accuracy, while UCP-LF CAA showed good sensitivity but limited specificity. The context-dependency of test performance extends beyond simple accuracy metrics to include practical considerations such as laboratory infrastructure requirements, technical expertise, and cost-effectiveness. While high sensitivity and specificity (≥ 90%) might be crucial in low-prevalence or elimination settings, lower accuracy thresholds could be acceptable in highly endemic areas focusing on MDA needs. However, the practical application of PCR in field settings is hampered by its significant infrastructure and human resource requirements. This study created the opportunity of strengthening local laboratory and diagnostic capacities showing the added value in the implementation of studies in endemic settings.

### Comparison with results from the literature

Our study extends the existing literature on the performance of laboratory tests for detecting schistosome infections. In the *S. mansoni* endemic region, our findings that POC-CCA has high Se and Sp, thereby facilitating acceptable infection classification, corroborate previous studies that reported variable performance of POC-CCA depending on endemicity and reference standards used [30]. In medium to high endemic areas where parasite burden is likely higher, POC-CCA has shown promising results, although persistence of very low reactivity (trace) in the absence of egg shedding has been observed [59, 60]. This is consistent with reports that the accuracy of POC-CCA is more robust in higher endemicity settings, although problems with trace results remain [30]. Our findings regarding UCP-LF CAA performance differ from previous studies that used composite reference standards or microscopy as reference, which typically reported higher specificity [32–34, 61]. However, our results align with other LCA, suggesting that the choice of reference standard significantly impacts performance estimates [35, 62, 63]. While UCP-LF CAA demonstrated high sensitivity in our study, the lower-than-expected specificity warrants further investigation. This finding is particularly relevant as the UCP-LF CAA test is mainly used for research purposes and it does not yet exist in a commercial format. Clinical trials are currently underway to evaluate the performances of an RDT format [64, 65]. PCR demonstrated high Se and Sp, consistent with its reported high performance in other studies [32, 36]. Importantly, PCR offers the additional advantage of being able to distinguish between *S. mansoni* and *S. haematobium* infections. While its application is limited by infrastructure and resource requirements, recent developments in molecular diagnostics for schistosomiasis, including loop-mediated isothermal amplification and recombinase polymerase amplification techniques, show promise for more field-applicable solutions [66]. In the *S. haematobium* endemic area, our results showed that POC-CCA had low Se and Sp (~ 60%), confirming the known caveats of its use for the detection of *S. haematobium* infections [67]. Furthermore, although UCP-LF CAA and PCR showed high Se, their imperfect Sp also compromised their diagnostic accuracy, highlighting the need for more reliable diagnostic methods for *S. haematobium*. Our results echo the recommendation of Coelho et al. to optimise POC-CCA in low worm burden samples [68], and highlight the ongoing debate on the interpretation of trace results. As shown by Prada et al. [69], trace results may still indicate active infection, highlighting the complexity of accurately diagnosing schistosomiasis post-treatment. The relationship between our diagnostic tests shows both similarities and differences to previous findings. The stronger correlations observed in *S. mansoni* endemic areas, particularly between POC-CCA and PCR (*ρ* = 0.60), align with previous studies that reported similar relationships between antigen levels and molecular markers. The relatively weaker correlations in *S. haematobium* areas (maximum *ρ* = 0.48) suggest more complex detection dynamics, possibly due to varying antigen excretion patterns or differential test sensitivities at different infection intensities.

### Strengths and Limitations

#### Strengths

First, we estimated the prevalence of two species of *Schistosoma*; understanding prevalence in a given healthcare setting is critical for planning and policy making [70–72]. Using BLCM, we have made the best possible use of data by including results from all available tests to determine Se and Sp, while accounting for the possibility of between-test dependence [52, 73, 74]. Second, our comprehensive dataset includes both qualitative diagnostic outcomes and quantitative measures of infection intensity through PCR Ct values, UCP-LF CAA concentrations, and semi-quantitative POC-CCA G-scores. This multi-dimensional assessment provides deeper insights into the relationship between test performance and infection intensity, which is crucial for understanding diagnostic accuracy across different endemicity settings. Third, data on diagnostic accuracy are often scarce for endemic populations, which have a different profile of infection intensity, host immunity, co-infections and environmental factors than the population in which they are initially validated. The collection of data from endemic regions is essential because diagnostic tests need to reflect real-world conditions in order to provide accurate and effective disease detection. Our study provides important insights into the diagnostic accuracy of different tests in rural Madagascar. Finally, a major strength is the comprehensive dataset from a large and diverse sample, which increases the reliability and applicability of our findings.

#### Limitations

First, as with any statistical model, it cannot be proven that the BLCM we fit are the true models. It is worth emphasizing that estimating diagnostic test accuracy is different from making a clinical decision. Here we constructed a model to estimate test performance and tried to be transparent about the unknowns, assumptions, and subjective choices, while other parameterisations are certainly possible. However, our models were reasonably well specified, as evidenced by the good agreement between observed and expected test result patterns and the low residual correlation between test results. While there is no ideal way to validate the results of an LCM as there is no perfect reference test, we attempted to validate our findings through sensitivity analyses using different prior specifications and trace result interpretations. However, due to the lack of additional external validation data such as the proportion of patients with a particular test pattern who were treated, our validation options were limited. Second, LCM has been described as a “black box” [75], and cautions have been raised that model misspecification is difficult to detect [76]. Certainly, its mechanisms are less intuitive to understand than Boolean decision rules as often used in defining CRS, but the theory underlying LCM is clearly defined and transparent [54, 77]. We have tried to be clear by providing a heuristic model that illustrate our assumptions about the relationships within the model. While our BLCM approach using various dependency structures appropriately captured the diagnostic uncertainty, other statistical methods could provide complementary insights. For instance, frequentist latent class models or Bayesian models with different prior specifications might offer additional perspectives on the test performance variations between endemic settings. However, given the consistent pattern of wider credible intervals for *S. haematobium* across our different model specifications, this uncertainty likely reflects true diagnostic challenges rather than methodological limitations. In addition, our diagnostic approach was developed with the input from a multidisciplinary team of experts to address potential model misspecification, as traditional goodness-of-fit metrics alone may not fully capture these issues. A final, general limitation related to the statistical model is that the parameter estimates depend on the available data which is true for any model estimate.

Due to the constraints of our study setting, which only allowed a single sample to be collected per participant, we were unable to include microscopy as a reference standard, despite its usual role as a key diagnostic tool to validate results. This limitation is relevant as microscopy’s sensitivity is known to improve with multiple samples, and its inclusion could have provided valuable comparative data. PCR's ability to identify active infections may be limited by biomarker persistence, though we mitigated this by excluding participants with PZQ treatment within 12 months [36]. Clinical variables that could influence diagnostic outcomes, such as infection intensity stage and disease stage, were unavailable. Our quantitative and semi-quantitative measures of infection intensity (PCR Ct values, UCP-LF CAA concentrations, and POC-CCA G-scores) have inherent limitations. POC-CCA is formally recommended only as a qualitative test with potential operator and batch variability, which we addressed through standardisation. The relationship between our measurements (PCR Ct values, UCP-LF CAA concentrations, POC-CCA G-scores) and actual worm burden remains incompletely validated. Finally, our use of consecutive sampling limits the generalisability of our findings to the broader population. While this sampling method was practical for our study setting, we recommend future studies employ probabilistic sampling methods to enable broader population-level inferences.

## Conclusions

Achieving the NTD roadmap target [4] to eliminate schistosomiasis as a public health problem in endemic countries hinges on the deployment of precise and sensitive diagnostic tests [42]. Our comprehensive evaluation of diagnostic tests in rural Madagascar, involving a large sample size across different endemic settings, provides robust evidence for test performance in real-world conditions. In *S. mansoni* endemic areas, the high sensitivity of POC-CCA combined with its field applicability makes it particularly suitable for initial prevalence assessment and program monitoring. However, in *S. haematobium* settings, the lower test agreement suggests that combining multiple diagnostic approaches might be necessary. Our results emphasize that it is essential to define the use case for each test and recommend its use according to the epidemiology, the context and the purpose. It also highlights the need to focus on underserved areas where data are scarce to generate valuable evidence that could guide better diagnostic practices and health policies in similar settings worldwide. These considerations align with recent calls for context-specific approaches to schistosomiasis diagnostics and highlight the need for flexible, evidence-based diagnostic strategies that can adapt to changing epidemiological situations. Advanced methods to overcome limitations in the assessment of test accuracy should be encouraged.

## Supplementary Information

Supplementary material 1

## Data Availability

The datasets used and/or analysed during the current study are available from the corresponding author on reasonable request.

## Abbreviations

BLCM: Bayesian latent class model
BNITM: Bernhard Nocht Institute for Tropical Medicine
CDP: Conditional dependence in positives
CDN: Conditional dependence in negatives
CDPN: Conditional dependence in positives and negatives
*CI*: Conditional independence
CrI: Credible interval
CRS: Composite reference standard
Ct: Cycle threshold
DALYs: Disability-adjusted life years
DNA: Deoxyribonucleic acid
IQR: Interquartile range
LCM: Latent class model
MDA: Mass drug administration
NTDs: Neglected tropical diseases
PABAK: Prevalence-adjusted and bias-adjusted kappa
PCR: Polymerase chain reaction
PHCC: Primary health care centre
POC-CCA: Point-of-care circulating cathodic antigen
PZQ: Praziquantel
RDTs: Rapid diagnostic tests
Se: Sensitivity
Sp: Specificity
SSA: Sub-Saharan African
UCP-LF CAA: Up-converting reporter particle lateral flow circulating anodic antigen
