# Supplementary material for: Detecting Schistosoma infections in endemic countries: a diagnostic accuracy study in rural Madagascar
Source: Infect Dis Poverty. 2025 Mar 17;14:20. doi: 10.1186/s40249-025-01292-x (PMC11912594; doi:10.1186/s40249-025-01292-x)
Supplement: Supplementary file 1 — Supplementary material 1 [file 40249_2025_1292_MOESM1_ESM.docx]

**Supplementary Information (SI):**

**SI 1. Descriptive statistics and diagnostic test agreements**

The study included 1339 participants from three PHCCs in Madagascar, with 461 participants from the *S. haematobium* endemic site and 878 from *S. mansoni* endemic sites. The sex distribution was similar across sites (54–55% female), but age distributions differed notably. Participants from the *S. haematobium* endemic site were generally younger (median age 28 years, *IQR*: 21–40) with more than half (53%) aged 18–29 years. In contrast, *S. mansoni* endemic sites showed a more even age distribution across all groups, with an older median age (38 years, *IQR*: 26–50) and the largest proportion (34%) in the 18–29 years age group.

**Table SI1.1.** Baseline demographic and treatment characteristics of participants from three PHCC in the provinces Mahajanga Ankazomborana, Tsironamandidy and Andina, Madagascar, who were interviewed and sampled within SCHISDIMA (total *n* = 1339; *S. haematobium* endemic area (MA), *n*: 461; *S. mansoni* endemic area (TO, FA), *n*: 878).

|  | ***S. haematobium* endemic site**  N: 461 | | ***S. mansoni* endemic sites**  N: 878 | |
| --- | --- | --- | --- | --- |
| **Characteristic** | ***n*** | **%**^c^ | ***n*** | **%**^c^ |
| **Sex^a^** |  |  |  |  |
| Female | 255 | 55 | 473 | 54 |
| **Age in years^b^** |  |  |  |  |
| Median (IQR) | 28 | (21, 40) | 38 | (26, 50) |
| Min–Max |  | 18 – 84 |  | 18 – 79 |
| **Age group^b^** |  |  |  |  |
| 18–29 years | 243 | 53 | 299 | 34 |
| 30–39 years | 90 | 20 | 166 | 19 |
| 40–49 years | 66 | 14 | 180 | 21 |
| ≥ 50 years | 62 | 13 | 233 | 27 |

^a^Missing sex information for 1 individual from MA, 2 individuals from TO, 1 individual from FA.

^b^Missing age information for 1 individual from MA, 3 individuals from TO, 1 individual from FA.

^c^Column contains percentages unless indicated otherwise.

*Abbreviations:* FA, Andina; IQR, Interquartile Range; MA, Ankazomborana; N, size of denominator; n, size of nominator; PHCC, primary health care centre; TO Tsironamandidy.

**Table SI1.2.** Proportion of positive test results expressed as percentages by each of the three diagnostic tests among the assessed individuals. Cumulative percentages may exceed 100 due to rounding imprecision.

|  | ***S. haematobium* endemic site**  N: 461 | | ***S. mansoni* endemic sites**  N: 878 | |
| --- | --- | --- | --- | --- |
|  | ***n*** | **Percentage [95% *CI*]** | ***n*** | **Percentage [95% *CI*]** |
| **POC-CCA positive** | 240 | 52 [47–57] | 476 | 54 [51–58] |
| Trace (G2-G3) | 95 | 21 [17–25] | 350 | 40 [37–43] |
| **UCP-LF CAA positive** | 275 | 60 [55–64] | 482 | 55 [52–58] |
| **PCR positive** | 305 | 66 [62–70] | 519 | 59 [56–62] |
| *S. haematobium* only | 281 | 61 [56–65] | 5 | 1 [0–1] |
| *S. mansoni* only | 8 | 2 [1–4] | 506 | 58 [54–61] |
| Both species | 16 | 4 [2–6] | 8 | 1 [0–2] |

*Abbreviations:* CI, confidence interval; N, size of denominator; n, size of nominator; PCR, polymerase chain reaction; POC-CCA, point-of-care circulating cathodic antigen; UCP-LF CAA, up-converting reporter particle lateral flow circulating anodic antigen.

The agreement between diagnostic tests varied substantially between endemic settings. In *S. mansoni* endemic sites, test agreement was generally better, with moderate agreement between POC-CCA and PCR (*κ* = 0.53, 95% *CI*: 0.48–0.59) and fair agreement for other test combinations (*κ* = 0.37). In contrast, the *S. haematobium* endemic site showed only slight to fair agreement between tests, with *κ* values ranging from 0.11 to 0.32 (Table SI1.3). The overall agreement across all three tests was moderate in *S. mansoni* endemic sites (Fleiss *κ* = 0.42, 95% *CI*: 0.39–0.46) but only slight in the *S. haematobium* endemic site (Fleiss *κ* = 0.19, 95% *CI*: 0.13–0.24). Notably, in the *S. haematobium* endemic site, the negative PABAK value (-0.18) for the three-test comparison suggests agreement worse than chance, indicating substantial systematic differences between test results in this setting.

**Table SI1.3.** Agreement of diagnostic tests quantified using Cohen’s Kappa to assess pairwise levels agreement and Fleiss Kappa coefficients to assess the overall agreement level across tests. Interpretation of kappa (*κ*): *κ* < 0: poor agreement; 0 < *κ* < 0.20: slight; 0.20 < *κ* < 0.40: fair; 0.40 < *κ* < 0.60: moderate; 0.60 < *κ* < 0.80: substantial. Agreements interpreted as fair and higher are shaded in lightgray. Negative PABAK values, as seen in the test comparison of all three tests in the *S. haematobium* endemic site, mean that the agreement between the tests is worse than would be expected by chance. This could indicate that in some cases the tests give very different results, which could indicate methodological differences or other systematic errors.

|  | ***S. haematobium* endemic site**  N: 461 | | ***S. mansoni* endemic sites**  N: 878 | |
| --- | --- | --- | --- | --- |
| **Test comparison** | **Interpretation** | **Cohen** *κ* **[95% *CI*]** | **Interpretation** | **Cohen** *κ* **[95% *CI*]** |
| POC-CCA and UCP-LF-CAA | slight | 0.11 [0.02–0.2] | fair | 0.37 [0.3–0.43] |
|  | **Percentage agreement / PABAK** | |  |  |
|  |  | 56% / 0.119 |  | 69% / 0.371 |
| POC-CCA and PCR | slight | 0.14 [0.06–0.23] | moderate | 0.53 [0.48–0.59] |
|  | **Percentage agreement / PABAK** | |  |  |
|  |  | 58% / 0.154 |  | 77% / 0.542 |
| UCP-LF CAA and PCR | fair | 0.32 [0.24–0.41] | fair | 0.37 [0.31–0.43] |
|  | **Percentage agreement / PABAK** | |  |  |
|  |  | 68% / 0.367 |  | 69% / 0.383 |
| **Test comparison** |  | **Fleiss** *κ* **[95% *CI*]** |  | **Fleiss** *κ* **[95% *CI*]** |
| All three tests | slight | 0.19 [0.13–0.24] | moderate | 0.42 [0.39–0.46] |
|  | **Percentage agreement / PABAK** | |  |  |
|  |  | 41% / -0.18 |  | 57% / 0.148 |

*Abbreviations:* CI, confidence interval; N, size of denominator; n, size of nominator; PABAK, Prevalence-Adjusted and Bias-Adjusted Kappa; PCR, polymerase chain reaction; POC-CCA, point-of-care circulating cathodic antigen; UCP-LF-CAA, up-converting reporter particle lateral flow circulating anodic antigen.

Table SI1.4 shows the pairwise cross-tabulation of all three diagnostic tests. The agreement between diagnostic tests varied by endemic setting. In *S. mansoni* endemic sites, the highest concordance was observed between POC-CCA and PCR, with 77% of samples showing matching results (32% both negative, 45% both positive). For *S. haematobium* endemic sites, the strongest agreement was found between UCP-LF-CAA and PCR, with 68% concordant results (21% both negative, 47% both positive). Notably, in the *S. mansoni* setting, POC-CCA showed substantial discordance with UCP-LF-CAA, where 16% of samples were POC-CCA negative but CAA positive, and 15% showed the inverse pattern. Similar discordance was observed in the *S. haematobium* setting (26% POC-CCA negative/CAA positive, 18% POC-CCA positive/CAA negative).

**Table SI1.4.** Pairwise cross-tabulation of diagnostic tests.

|  |  | ***S. haematobium* endemic site**  N: 461 | | ***S. mansoni* endemic sites**  N: 878 | |
| --- | --- | --- | --- | --- | --- |
|  |  | **UCP-LF CAA** |  | **UCP-LF CAA** |  |
| **POC-CCA** |  | **negative** | **positive** | **negative** | **positive** |
|  | **negative** | 102 (22%) | 119 (26%) | 261 (30%) | 141 (16%) |
|  | **positive** | 84 (18%) | 156 (34%) | 135 (15%) | 341 (39%) |
|  |  | **PCR** |  | **PCR** |  |
| **POC-CCA** |  | **negative** | **positive** | **negative** | **positive** |
|  | **negative** | 91 (20%) | 130 (28%) | 280 (32%) | 122 (14%) |
|  | **positive** | 65 (14%) | 175 (38%) | 79 (9%) | 397 (45%) |
|  |  | **PCR** |  | **PCR** |  |
| **UCP-LF CAA** |  | **negative** | **positive** | **negative** | **positive** |
|  | **negative** | 98 (21%) | 88 (19%) | 242 (28%) | 154 (18%) |
|  | **positive** | 58 (13%) | 217 (47%) | 117 (13%) | 365 (42) |

*Abbreviations:* N, size of denominator; PCR, polymerase chain reaction; POC-CCA, point-of-care circulating cathodic antigen; UCP-LF-CAA, up-converting reporter particle lateral flow circulating anodic antigen.

When using a composite reference standard (CRS) of either PCR or UCP-LF CAA positivity, concordance with POC-CCA varied across endemic settings (Table SI1.5). In the *S. haematobium* endemic site, POC-CCA showed lower agreement (55% concordant results), with a substantial proportion (36%) of cases being CRS-positive but POC-CCA negative. In *S. mansoni* endemic sites, POC-CCA demonstrated higher agreement with the CRS, with 71% concordant results (22% both negative, 49% both positive).

**Table SI1.5.** Cross-tabulation of diagnostic tests when PCR and UCP-LF CAA are considered a composite reference standard with test results being interpreted as positive if either PCR or UCP-LF CAA or both tests are positive [85].

|  |  | ***S. haematobium* endemic site**  N: 461 | | ***S. mansoni* endemic sites**  N: 878 | |
| --- | --- | --- | --- | --- | --- |
|  |  | **CRS (PCR and/or CAA positive)** | | **CRS (PCR and/or CAA positive)** | |
| **POC-CCA** |  | **negative** | **positive** | **negative** | **positive** |
|  | **negative** | 54 (12%) | 167 (36%) | 195 (22%) | 207 (24%) |
|  | **positive** | 44 (10%) | 196 (43%) | 47 (5%) | 429 (49%) |

*Abbreviations:* N, size of denominator; PCR, polymerase chain reaction; POC-CCA, point-of-care circulating cathodic antigen; UCP-LF-CAA, up-converting reporter particle lateral flow circulating anodic antigen.

The CSR analysis provides additional insights beyond the pairwise comparisons. In the *S. haematobium* endemic site, where pairwise agreements were only slight (*κ* = 0.11–0.14), with POC-CCA missing over one-third of CRS-positive cases. For *S. mansoni*, despite showing better pairwise agreements (moderate with PCR, *κ* = 0.53; fair with CAA, *κ* = 0.37), POC-CCA still demonstrated room for improvement with. These findings suggest that while POC-CCA performs better in *S. mansoni* endemic areas, its diagnostic accuracy might be insufficient as a standalone test, particularly in *S. haematobium* settings where it misses a considerable proportion of CRS-positive cases.

In the *S. haematobium* endemic region, the strongest correlation was found between UCP-LF CAA and PCR (*ρ* = 0.48, 95% *CI*: 0.36–0.58), followed by POC-CCA and PCR (*ρ* = 0.41, 95% *CI*: 0.28–0.52), and POC-CCA and UCP-LF CAA (*ρ* = 0.40, 95% *CI*: 0.28–0.52). In the *S. mansoni* endemic region, the strongest correlation was found between POC-CCA and PCR (*ρ* = 0.60, 95% *CI*: 0.53–0.66), followed by POC-CCA and UCP-LF AG (*ρ* = 0.52, 95% *CI*: 0.44–0.59), and UCP-LF CAA and PCR (*ρ* = 0.44, 95% *CI*: 0.35–0.52).

**SI 2. Bayesian latent class models (BLCM)**

**2.1 BLCM Priors – rationale**

Initially non-informative priors were used for all models (beta distribution with *α* = 1 and *β* = 1) with truncated distributions for the non-specific tests’ Se and Sp to contain them above 40% and avoid label switching (mirror solutions).

To complement these findings, we ran models with informative priors based on estimates from previous studies. Priors for model Sp for PCR were set to follow a beta-distribution with *α* = 9 and *β* = 3, i.e., centred around 90% and ranging from 60–100% [53]. Priors for Sp for UCP-LF CAA were set to follow a beta-distribution with *α* = 18 and *β* = 3, i.e., centred around 90% and ranging from 75–100% [65, 66, 69, 86].

**2.2 Results on models with non-informative priors**

The detailed model estimates (Tables SI2.1 and SI2.2) confirm the robustness of our findings across different model specifications. For both endemic settings, point estimates and credible intervals showed high consistency across all dependency structures (Models 0–4).

**Table SI2.1.** Model estimates for sensitivity, specificity, PPV, and NPV per diagnostic test and LCA model and overall prevalence based on diagnostic test results from 461 participants from *S. haematobium* endemic site.

| **Test** | **Characteristic** | **Model estimates (point estimate [95% Credible Interval])** | | | | |
| --- | --- | --- | --- | --- | --- | --- |
|  |  | **Model 0** | **Model 1** | **Model 2** | **Model 3** | **Model 4** |
| ***S. haematobium* endemic area** | | | | | | |
| POC-CCA | Sensitivity | 64.5  [55.5–74.5] | 62.32  [53.63–73.35] | 61.8  [53.39–72.71] | 63.88  [54.73–74.29] | 61.84  [53.28–72.67] |
| POC-CCA | Specificity | 59.57  [52.06–68.07] | 59.05  [50.57–67.8] | 59.04  [50.85–67.46] | 59.36  [51.59–67.98] | 58.74  [50.63–67.11] |
| POC-CCA | PPV | 60.01  [44.94–77.29] | 62.27  [45.76–79.79] | 63.35  [46.2–79.91] | 60.35  [44.83–78.73] | 62.63  [45.31–79.69] |
| POC-CCA | NPV | 64.66  [40.36–80.07] | 59.81  [34.51–78.67] | 58  [33.71–77.94] | 63.71  [38.09–79.74] | 58.68  [34–77.88] |
| UCP-LF CAA | Sensitivity | 87.78  [73.17–99.13] | 84.01  [69.96–98.83] | 82.73  [69.71–98.47] | 87.24  [72.64–99.07] | 83.56  [69.91–98.58] |
| UCP-LF CAA | Specificity | 66.19  [56.76–82.85] | 65.98  [56.55–81.77] | 65.94  [56.65–81.96] | 66.16  [56.51–86.38] | 65.9  [56.48–83.55] |
| UCP-LF CAA | PPV | 70.78  [55.12–89.7] | 72.89  [56.02–90.19] | 73.64  [56.63–90.58] | 71.3  [55.04–92.79] | 73.45  [56.5–91.43] |
| UCP-LF CAA | NPV | 85.58  [57.32–99.13] | 79.67  [49.19–98.85] | 77.62  [48.54–98.55] | 84.88  [55.29–99.08] | 78.96  [49.15–98.63] |
| PCR | Sensitivity | 95.24  [83.07–99.77] | 95.46  [83.41–99.81] | 95.41  [83.21–99.78] | 95.17  [81.79–99.81] | 95.46  [82.8–99.83] |
| PCR | Specificity | 60.31  [48.81–84.36] | 64.22  [49.39–96.13] | 65.68  [49.57–96.4] | 60.68  [48.62–86.15] | 64.64  [49.43–96.29] |
| PCR | PPV | 68.82  [50.92–91.86] | 73.79  [52.29–98.19] | 75.62  [52.77–98.37] | 69.61  [51.29–93.17] | 74.52  [52.36–98.25] |
| PCR | NPV | 93.39  [68.8–99.72] | 93.31  [68.35–99.75] | 93.06  [67.12–99.7] | 93.25  [64.83–99.76] | 93.2  [66.85–99.77] |
|  | Prevalence | 48.07  [34.38–67.82] | 51.63  [35.3–72.01] | 53.06  [35.74–72.62] | 48.65  [34.63–69.77] | 52.29  [35.48–72.26] |

**Table SI2.2.** Model estimates for sensitivity, specificity, PPV, and NPV per diagnostic test and LCA model and overall prevalence based on diagnostic test results from 878 participants from *S. mansoni* endemic sites.

| **Test** | **Characteristic** | **Model estimates (point estimate [95% Credible Interval])** | | | | |
| --- | --- | --- | --- | --- | --- | --- |
|  |  | **Model 0** | **Model 1** | **Model 2** | **Model 3** | **Model 4** |
| ***S. mansoni* endemic area** | | | | | | |
| POC-CCA | Sensitivity | 90.61  [85.02–96.31] | 88.26  [76.96–95.56] | 88.51  [77.22–95.54] | 90.49  [85.11–96.28] | 87.88  [76.6–95.33] |
| POC-CCA | Specificity | 81.54  [75.89–87.06] | 81.44  [75.86–87.04] | 81.54  [75.94–87.11] | 80.69  [75.05–86.52] | 80.75  [75.13–86.52] |
| POC-CCA | PPV | 82.8  [76.2–88.73] | 83.56  [76.74–89.56] | 83.59  [76.84–89.58] | 82.58  [76.01–88.63] | 83.58  [76.73–89.49] |
| POC-CCA | NPV | 89.81  [82.73–96.27] | 86.89  [69.83–95.48] | 87.17  [70.01–95.51] | 89.92  [83.15–96.34] | 86.66  [69.6–95.35] |
| UCP-LF CAA | Sensitivity | 79.91  [75.24–84.58] | 78.16  [70.05–83.67] | 78.36  [70.18–83.74] | 79.86  [74.99–84.46] | 77.87  [69.59–83.52] |
| UCP-LF CAA | Specificity | 69.72  [64.17–75.2] | 69.71  [64.1–75.06] | 69.69  [64.2–75.21] | 69.1  [63.65–74.61] | 69.14  [63.66–74.71] |
| UCP-LF CAA | PPV | 72.14  [64.96–78.66] | 73.43  [65.52–80.47] | 73.29  [65.61–80.4] | 71.86  [64.58–78.44] | 73.34  [65.51–80.4] |
| UCP-LF CAA | NPV | 77.99  [71.32–84.01] | 75.43  [60.14–82.95] | 75.61  [60.45–83] | 78.32  [71.67–84.12] | 75.31  [60.19–82.97] |
| PCR | Sensitivity | 95.66  [91.03–99.46] | 95.68  [91–99.43] | 95.64  [90.96–99.47] | 96.55  [91.35–99.81] | 96.45  [91.41–99.78] |
| PCR | Specificity | 76.74  [70.2–83.16] | 79.37  [70.94–96.91] | 79.06  [71.05–96.65] | 76.87  [70.24–83.17] | 79.75  [71.28–97.75] |
| PCR | PPV | 80.13  [72.78–86.74] | 82.88  [73.64–97.91] | 82.64  [73.6–97.76] | 80.07  [72.54–86.48] | 83.19  [73.84–98.47] |
| PCR | NPV | 94.73  [88.57–99.38] | 94.52  [87.85–99.33] | 94.51  [87.67–99.38] | 95.87  [88.98–99.78] | 95.55  [88.51–99.73] |
|  | Prevalence | 49.5  [43.7–55.44] | 51.43  [44.35–61.22] | 51.24  [44.38–61.15] | 49.03  [43.25–54.99] | 51.25  [44.24–61.25] |

To systematically compare test performances while accounting for uncertainty, we examined the overlap of credible intervals between tests. For *S. mansoni*, PCR demonstrated higher sensitivity than UCP-LF CAA (95% *CI*: 91.0-99.5 vs 75.2-84.6), while POC-CCA showed intermediate performance (85.0-96.3). Specificity comparisons revealed substantial overlap between tests, suggesting no significant differences. For *S. haematobium*, the overlapping credible intervals for both sensitivity and specificity indicate no differences between tests, though point estimates suggest potential performance differences.

Table SI2.3 shows the model comparison based on expected log predictive density (ELPD), with each model evaluated against the model with the highest ELPD. For each model, the ELPD difference indicates the difference in the Bayesian leave-one-out cross-validated estimates of the expected log pointwise predictive density (elpd_loo), while the SE difference reflects the standard error of the component-wise differences in elpd_loo. Model comparison based on expected log predictive density (Table SI2.3) showed minimal differences between all models (ELPD differences < 1), with correspondingly small standard errors of these differences (SE < 0.5). This suggests that different assumptions about conditional dependence between tests had little impact on model performance, supporting the hypothesis that the correlations are not significant.

**Table SI2.3.** Model comparison on expected log predictive density.

| **Model** | **ELPD difference^a^** | **SE^b^ difference** |
| --- | --- | --- |
| ***S. haematobium* endemic area** | | |
| Model 4 | -0.4 | 0.5 |
| Model 3 | -0.7 | 0.2 |
| Model 2 | -0.2 | 0.3 |
| Model 1 | -0.1 | 0.4 |
|  | **ELPD** | **SE** |
| Model 0 | -898.3 | 11.5 |
| ***S. mansoni* endemic area** |  |  |
| Model 4 | -0.6 | 0.3 |
| Model 3 | -0.1 | 0.3 |
| Model 2 | -0.2 | 0.2 |
| Model 1 | -0.2 | 0.1 |
|  | **ELPD** | **SE** |
| Model 0 | -1592.3 | 21.4 |

^a^ Expected log predictive density difference is the difference in Bayesian leave one out estimate of the expected log pointwise predictive density between two models (elpd_loo). Comparison is made between each model and the model with the largest expected log predictive density.

^b^ Standard error of component-wide differences in elpd_loo.

**2.3 Results on models with informative priors for UCP-LF CAA and PCR specificity**

Using informative priors led to slightly higher prevalence estimates for *S. haematobium* (57.4-64.5%) while estimates remained stable for *S. mansoni* (50.2-51.8%). Diagnostic accuracy estimates showed modest improvements in Sp for both UCP-LF CAA and PCR, as expected given the informative priors, while Se estimates remained largely unchanged. Model comparison again showed minimal differences between models (ELPD differences ≈ 1).

**Table SI2.4.** Model estimates for sensitivity, specificity, PPV, and NPV per diagnostic test and LCA model and overall prevalence based on diagnostic test results from 461 participants from *S. haematobium* endemic area using informative priors for UCP-LF CAA and PCR specificity.

| **Test** | **Characteristic** | **Model estimates (point estimate [95% Credible Interval])** | | | | |
| --- | --- | --- | --- | --- | --- | --- |
|  |  | **Model 0** | **Model 1** | **Model 2** | **Model 3** | **Model 4** |
| ***S. haematobium* endemic area** | | | | | | |
| POC-CCA | Sensitivity | 61.35  [53.33–70.76] | 59.82  [52.19–69.14] | 59.61  [52.25–68.75] | 59.86  [52.47–68.03] | 58.43  [51.27–66.88] |
| POC-CCA | Specificity | 60.38  [51.89–69.78] | 59.43  [50.07–68.79] | 59.49  [50.13–68.84] | 60.89  [51.38–70.3] | 59.38  [48.58–69.23] |
| POC-CCA | PPV | 67.93  [51.35–82.98] | 69.16  [51.84–83.11] | 69.65  [52.33–83.35] | 72.31  [57.53–84.83] | 72.51  [57.56–84.64] |
| POC-CCA | NPV | 53.8  [30.86–73.13] | 49.54  [28.2–71] | 48.8  [27.76–70.21] | 47.65  [27.95–65.49] | 44.06  [25.27–64.08] |
| UCP-LF CAA | Sensitivity | 83.56  [71.74–98] | 80.29  [70.36–96.93] | 80.03  [70.16–96.89] | 81.09  [71.08–93.4] | 79.01  [70.03–92.09] |
| UCP-LF CAA | Specificity | 73.89  [62.31–91.52] | 73.27  [61.83–91.16] | 73.59  [62.27–91.32] | 77.58  [69.96–92.94] | 77.06  [69.92–92.86] |
| UCP-LF CAA | PPV | 81.37  [64.66–96.01] | 81.9  [65.88–95.96] | 82.41  [66.43–96.15] | 86.35  [74.43–97.16] | 86.66  [74.8–97.29] |
| UCP-LF CAA | NPV | 77.01  [50.69–97.8] | 71.12  [47.9–96.63] | 70.23  [47.04–96.58] | 71.26  [47.68–91.78] | 67.21  [45.57–90.24] |
| PCR | Sensitivity | 90.23  [79.06–99.26] | 90.85  [79.51–99.44] | 90.45  [79.31–99.29] | 87.56  [78.32–97.41] | 87.88  [78.21–97.86] |
| PCR | Specificity | 66.82  [52.68–88.09] | 71.23  [54.04–91.72] | 71.9  [53.81–91.98] | 70.28  [60.67–89.18] | 73.79  [60.94–92.18] |
| PCR | PPV | 78.73  [59.34–95.13] | 82.95  [61.39–96.54] | 83.54  [61.7–96.68] | 83.32  [69.7–95.82] | 86.2  [70.47–97.02] |
| PCR | NPV | 83.77  [57.11–98.95] | 84.16  [56.35–99.2] | 83.43  [55.68–98.93] | 77.32  [53.56–96.15] | 77.35  [52.98–96.62] |
|  | Prevalence | 57.41  [40.58–75.4] | 60.13  [42.19–76.59] | 60.79  [42.5–77.1] | 62.72  [47.67–77.53] | 64.51  [48.44–78.48] |

**Table SI2.5.** Model estimates for sensitivity, specificity, PPV, and NPV per diagnostic test and LCA model and overall prevalence based on diagnostic test results from 878 participants from *S. mansoni* endemic area using informative priors for UCP-LF CAA and PCR specificity.

| **Test** | **Characteristic** | **Model estimates (point estimate [95% Credible Interval])** | | | | |
| --- | --- | --- | --- | --- | --- | --- |
|  |  | **Model 0** | **Model 1** | **Model 2** | **Model 3** | **Model 4** |
| ***S. mansoni* endemic area** | | | | | | |
| POC-CCA | Sensitivity | 90.04  [84.75–95.37] | 88.01  [78.9–94.88] | 88.34  [79.33–94.62] | 89.52  [84.38–94.46] | 87.77  [78.87–93.66] |
| POC-CCA | Specificity | 82.00  [76.39–87.58] | 81.93  [76.16–87.41] | 81.9  [76.34–87.24] | 81.38  [75.65–87.04] | 81.36  [75.73–87.1] |
| POC-CCA | PPV | 83.47  [76.96–89.27] | 84.02  [77.38–89.73] | 83.9  [77.52–89.49] | 83.7  [77.46–89.43] | 84.28  [77.99–89.86] |
| POC-CCA | NPV | 89.09  [82.22–95.28] | 86.53  [73.02–94.69] | 86.95  [73.58–94.48] | 88.64  [82.13–94.29] | 86.43  [73.4–93.53] |
| UCP-LF CAA | Sensitivity | 79.84  [75.01–84.33] | 78.3  [71.27–83.72] | 78.5  [71.58–83.68] | 79.76  [75.03–84.32] | 78.34  [71.5–83.57] |
| UCP-LF CAA | Specificity | 71.11  [65.76–76.25] | 71.02  [65.72–76.23] | 71.05  [65.66–76.27] | 71.58  [68.29–76.19] | 71.54  [68.22–76.1] |
| UCP-LF CAA | PPV | 73.65  [66.63–79.69] | 74.55  [67.12–80.76] | 74.39  [67.14–80.66] | 74.56  [69.5–80.02] | 75.45  [69.95–81.25] |
| UCP-LF CAA | NPV | 77.79  [71.07–83.68] | 75.5  [63.28–82.83] | 75.84  [63.84–82.79] | 78.11  [71.56–83.94] | 76.08  [64.32–82.99] |
| PCR | Sensitivity | 95.18  [90.57–99.17] | 95.21  [90.62–99.25] | 95.21  [90.61–99.24] | 95.79  [90.85–99.64] | 95.79  [90.83–99.64] |
| PCR | Specificity | 77.41  [71.08–83.48] | 79.58  [71.95–92.32] | 79.2  [71.81–91.68] | 77.93  [71.87–83.93] | 79.87  [72.45–92.2] |
| PCR | PPV | 81.00  [73.87–87.07] | 83.27  [74.8–94.68] | 82.88  [74.74–94.16] | 81.38  [74.79–87.25] | 83.42  [75.65–94.58] |
| PCR | NPV | 94.08  [87.85–99.05] | 93.93  [87.33–99.11] | 93.98  [87.49–99.11] | 94.84  [88.07–99.58] | 94.67  [87.66–99.58] |
|  | Prevalence | 50.24  [44.4–55.85] | 51.82  [45.08–59.93] | 51.57  [45.06–59.63] | 50.19  [44.71–55.8] | 51.66  [45.49–59.68] |

**Table SI2.6.** Model comparison on expected log predictive density using informative priors for UCP-LF CAA and PCR specificity.

| **Model** | **ELPD difference^a^** | **SE^b^ difference** |
| --- | --- | --- |
| ***S. haematobium* endemic area** | | |
| Model 4 | -0.9 | 0.8 |
| Model 3 | -1.1 | 0.5 |
| Model 2 | -0.1 | 0.6 |
| Model 1 | -0.2 | 0.6 |
| Model 0 | -899.6 | 11.6 |
| ***S. mansoni* endemic area** |  |  |
| Model 4 | -0.1 | 0.6 |
| Model 3 | -0.1 | 0.6 |
| Model 2 | -0.3 | 0.2 |
| Model 1 | -0.2 | 0.2 |
| Model 0 | -1592.4 | 21.6 |

^a^ Expected log predictive density difference is the difference in Bayesian leave one out estimate of the expected log pointwise predictive density between two models (elpd_loo). Comparison is made between each model and the model with the largest expected log predictive density.

^b^ Standard error of component-wide differences in elpd_loo.

**2.4 Results on models with non-informative priors and POC-CCA trace results interpreted as positive**

This approach notably impacted prevalence estimates, particularly for *S. mansoni* (increased to 77.4–77.7%) and moderately for *S. haematobium* (55.7–59.3%). POC-CCA Se increased substantially while Sp decreased, as expected when reclassifying trace results. Other test characteristics remained relatively stable. Model comparison continued to show minimal differences between dependency structures.

**Table SI2.4.** Model estimates for sensitivity, specificity, PPV, and NPV per diagnostic test and LCA model and overall prevalence based on diagnostic test results from 461 participants from *S. haematobium* endemic area using non-informative priors and interpreting POC-CCA trace results as positive.

| **Test** | **Characteristic** | **Model estimates (point estimate [95% Credible Interval])** | | | | |
| --- | --- | --- | --- | --- | --- | --- |
|  |  | **Model 0** | **Model 1** | **Model 2** | **Model 3** | **Model 4** |
| ***S. haematobium* endemic area** | | | | | | |
| POC-CCA | Sensitivity | 80.93  [73.77–89.06] | 79.89  [72.95–88.48] | 79.53  [72.73–88.07] | 80.7  [73.26–88.94] | 79.63  [72.76–88.13] |
| POC-CCA | Specificity | 41.77  [40.07–48.03] | 41.7  [40.08–47.7] | 41.72  [40.07–47.69] | 41.92  [40.05–48.12] | 41.83  [40.04–47.57] |
| POC-CCA | PPV | 63.88  [47.92–80.93] | 65.69  [48.28–81.45] | 66.7  [48.99–81.97] | 64.48  [47.88–81.84] | 66.35  [48.58–82.12] |
| POC-CCA | NPV | 64.04  [36.35–84.46] | 60.42  [35.35–83.56] | 58.72  [33.73–82.37] | 62.92  [34.25–83.92] | 59.22  [33.45–82.77] |
| UCP-LF CAA | Sensitivity | 82.55  [70.31–96.77] | 80.34  [69.15–96.59] | 79.44  [69.07–95.78] | 82.24  [70.11–96.71] | 79.93  [69.09–96.12] |
| UCP-LF CAA | Specificity | 69.14  [57.83–89.3] | 68.51  [57.65–87.99] | 68.82  [57.82–88.28] | 69.36  [57.82–92.04] | 68.9  [57.69–90.41] |
| UCP-LF CAA | PPV | 77.29  [60.2–95] | 78.03  [60.39–94.66] | 78.84  [61.25–94.9] | 77.98  [59.98–96.76] | 78.98  [60.8–96.16] |
| UCP-LF CAA | NPV | 76.14  [47.75–96.62] | 71.96  [45.58–96.54] | 69.96  [44.25–95.46] | 75.41  [46.83–96.6] | 70.97  [44.96–95.98] |
| PCR | Sensitivity | 91.71  [79.72–99.47] | 92.21  [80.35–99.5] | 91.91  [80.23–99.5] | 91.48  [78.85–99.44] | 91.9  [79.6–99.52] |
| PCR | Specificity | 65.79  [51.07–92.74] | 68.95  [51.35–97.23] | 70.58  [51.9–97.31] | 66.12  [51–93.11] | 69.87  [51.84–96.81] |
| PCR | PPV | 77.24  [56.51–97.08] | 80.45  [57.01–98.84] | 82.07  [57.83–98.9] | 77.93  [56.37–97.33] | 81.51  [57.68–98.73] |
| PCR | NPV | 86.7  [57.31–99.27] | 87.03  [58.42–99.31] | 86.29  [57.37–99.23] | 86.04  [54.66–99.21] | 86.29  [56.32–99.3] |
|  | Prevalence | 55.71  [38.77–75.63] | 58.1  [39.29–76.59] | 59.33  [39.98–77.25] | 56.48  [38.64–76.87] | 58.82  [39.58–77.45] |

**Table SI2.5.** Model estimates for sensitivity, specificity, PPV, and NPV per diagnostic test and LCA model and overall prevalence based on diagnostic test results from 878 participants from *S. mansoni* endemic area using non-informative priors and interpreting POC-CCA trace results as positive.

| **Test** | **Characteristic** | **Model estimates (point estimate [95% Credible Interval])** | | | | |
| --- | --- | --- | --- | --- | --- | --- |
|  |  | **Model 0** | **Model 1** | **Model 2** | **Model 3** | **Model 4** |
| ***S. mansoni* endemic area** | | | | | | |
| POC-CCA | Sensitivity | 99.29  [97.82–99.95] | 99.18  [97.71–99.94] | 99.18  [97.66–99.94] | 99.28  [97.73–99.95] | 99.17  [97.65–99.93] |
| POC-CCA | Specificity | 40.51  [40.02–42.77] | 40.5  [40.02–42.65] | 40.51  [40.02–42.72] | 40.59  [39.97–42.84] | 40.58  [39.97–42.82] |
| POC-CCA | PPV | 85.17 [  81.34–88.82] | 85.22  [81.52–88.89] | 85.25  [81.5–89.01] | 85.19  [81.37–88.94] | 85.31  [81.51–89.01] |
| POC-CCA | NPV | 94.43  [82.38–99.64] | 93.55  [81.55–99.53] | 93.53  [81.05–99.56] | 94.23  [81.6–99.57] | 93.45  [81.1–99.47] |
| UCP-LF CAA | Sensitivity | 67.71  [63.12–72.2] | 67.54  [63.02–72.11] | 67.48  [63.03–71.96] | 67.65  [62.95–72.29] | 67.51  [63.02–72.12] |
| UCP-LF CAA | Specificity | 89.05  [81.93–94.99] | 88.91  [81.64–94.8] | 88.9  [81.69–94.7] | 89.07  [81.78–95.13] | 88.89  [81.6–94.74] |
| UCP-LF CAA | PPV | 95.52  [92.03–98.15] | 95.5  [92–98.06] | 95.48  [91.96–98.04] | 95.6  [92.06–98.23] | 95.56  [92.2–98.08] |
| UCP-LF CAA | NPV | 44.48  [34.88–53.67] | 44.27  [34.27–53.26] | 44.11  [34.08–53.35] | 44.51  [34.68–53.77] | 44.08  [34.02–53.47] |
| PCR | Sensitivity | 74.24  [69.46–79.02] | 74.35  [69.48–79.14] | 74.33  [69.38–79.06] | 74.25  [69.2–79] | 74.29  [69.38–79.05] |
| PCR | Specificity | 93.01  [86.03–98.46] | 93.66  [86.17–99.34] | 93.69  [86.08–99.3] | 92.98  [85.93–98.61] | 93.7  [86.36–99.27] |
| PCR | PPV | 97.33  [94.25–99.49] | 97.59  [94.38–99.77] | 97.61  [94.34–99.77] | 97.34  [94.22–99.52] | 97.62  [94.54–99.75] |
| PCR | NPV | 51.26  [40.39–61.48] | 51.34  [40.5–61.5] | 51.31  [40.03–61.45] | 51.18  [39.85–61.42] | 51.16  [40.02–61.26] |
|  | Prevalence | 77.43  [72.3–82.59] | 77.55  [72.54–82.7] | 77.57  [72.47–82.9] | 77.47  [72.31–82.75] | 77.65  [72.52–82.82] |

**Table SI2.6.** Model comparison on expected log predictive density using informative priors for UCP-LF CAA and PCR specificity.

| **Model** | **ELPD difference^a^** | **SE^b^ difference** |
| --- | --- | --- |
| ***S. haematobium* endemic area** | | |
| Model 4 | -0.3 | 0.4 |
| Model 3 | -0.6 | 0.2 |
| Model 2 | -0.4 | 0.3 |
| Model 1 | -0.1 | 0.3 |
| Model 0 | -854.1 | 15.1 |
| ***S. mansoni* endemic area** |  |  |
| Model 4 | -0.3 | 0.2 |
| Model 3 | -0.6 | 0.1 |
| Model 2 | -0.6 | 0.3 |
| Model 1 | -0.2 | 0.2 |
| Model 0 | -1339.5 | 20.3 |

^a^ Expected log predictive density difference is the difference in Bayesian leave one out estimate of the expected log pointwise predictive density between two models (elpd_loo). Comparison is made between each model and the model with the largest expected log predictive density.

^b^ Standard error of component-wide differences in elpd_loo.

Across all three approaches, the consistency of estimates between different dependency structures (Models 0–4) supports the robustness of our findings. While the choice of priors had modest effects, the interpretation of trace results emerged as the most influential factor, particularly affecting prevalence estimates and POC-CCA performance metrics.

**2.5 BLCM Implementation Code**

The R code implementing the Bayesian Latent Class Models described in sections 2.1–2.4, including data pre-processing, model specifications, MCMC estimation using Stan, and diagnostic calculations, is provided at GitHub repository: https://github.com/evalorenz/SCHISDIMA_Code_R_Stan. The code includes implementation of the model variants across the two endemicity settings.
